# Experimental Studies on Durability of PVD-Based CrCN/CrN-Coated Cutting Blade of Planer Knives Used in the Pine Wood Planing Process

**DOI:** 10.3390/ma13102398

**Published:** 2020-05-22

**Authors:** Krzysztof Nadolny, Wojciech Kapłonek, Marzena Sutowska, Paweł Sutowski, Piotr Myśliński, Adam Gilewicz

**Affiliations:** 1 Department of Production Engineering, Faculty of Mechanical Engineering, Koszalin University of Technology, Racławicka 15-17, 75-620 Koszalin, Poland; wojciech.kaplonek@tu.koszalin.pl (W.K.); marzena.sutowska@tu.koszalin.pl (M.S.); pawel.sutowski@tu.koszalin.pl (P.S.); 2 Department of Technical Physics and Nanotechnology, Faculty of Mechanical Engineering, Koszalin University of Technology, Racławicka 15-17, 75-620 Koszalin, Poland; piotr.myslinski@tu.koszalin.pl; 3 Department of Bioengineering, Faculty of Mechanical Engineering, Koszalin University of Technology, Racławicka 15-17, 75-620 Koszalin, Poland; adam.gilewicz@tu.koszalin.pl

**Keywords:** antiwear PVD-based coating, industrial planer knife, woodworking, planing process, wear, durability

## Abstract

The condition of the cutting tool is one of the most important factors as it directly affects the technological and economic efficiencies of the woodworking process. The large variety of raw materials of wood combined with possible impurities and inclusion of solids puts high demands on planing machines. One of the methods to modify their operational properties is to apply antiwear coating on their working surfaces using vacuum deposition methods, such as physical vapor deposition (PVD). The use of such coatings reduces the adhesion of planing products to the surface of industrial planer knives, reduces the friction between the cutting tool and the workpiece, and limits the penetration of heat into the tool, thereby contributing to extending its effective working life. This study examines the impact of PVD-based CrCN/CrN coating on the operational durability and intensity of wear of planer knives operating in production conditions compared to unmodified knives (typically used in the wood processing industry for pine wood planing). For the unmodified and CrCN/CrN-coated planing blades (before and after processing), detailed analyses were carried out. These analyses included determining the rounding radius and profile along the blade (worn edge displacement), calculating surface texture parameters of the rake face of planer knives, and carrying out visual microscopic analysis of its condition. The results of the experiments indicated an increase in durability of up to 142% for the CrCN/CrN-coated tools. It was also found that the use of PVD-based modified industrial planer knives turned out to be more beneficial in each analyzed area of analysis.

## 1. Introduction

The wood and paper industry is one of the world’s leading processing industries. It is especially well developed in countries that extensively explore their own wood resources, such as the United States, Canada, Germany, China, Finland, Sweden, Russia, and Brazil, as widely described by Hansen et al. [1], Blennow and Niklasson [2], and Lele et al. [3]. The sawmill industry, characterized by Walker [4], is one of the branches of the wood industry related to the initial phase of processing of wood stock, i.e., from the separation of material into assortments to their saturation with impregnating substances. The first phase of stock processing is important because it determines the subsequent quality, size (dimensions), and usage of a given wood product range. Therefore, the greatest attention is paid to its proper machining.

One of the most important factors affecting the technological and economic efficiencies of the woodworking process is the condition of the cutting tools, which was analyzed in the works of Csanády and Magoss [5], Spinelli et al. [6], Ghosh et al. [7], Fahrussiam et al. [8], Twardowski et al. [9], Maruda et al. [10], and Krolczyk et al. [11] among others. The present study focuses on the planing process of pine wood, specifically the Scots pine (*Pinus sylvestris* L.) in the wet (not dried) condition used for producing small architectural objects, such as in gardening (gazebos, garden houses, pergolas, tables and benches, swings, etc.). The stock in the form of beams must be machined in all sides with high machine efficiency (planing speed of ~60 m/min) and with the required quality standards. Therefore, specialized multispindle planers with knife and mill assemblies mounted on the cutter heads are used for machining. The large variety of wood stock structure combined with possible impurities and inclusions of solids puts high demands on planing tools, as reported in the works of Nouveau et al. [12] and Soury et al. [13]. One of the ways to modify their operational properties is to apply antiwear coatings on their working surfaces using vacuum deposition methods, such as physical vapor deposition (PVD) and chemical vapor deposition (CVD), as presented in the works of Fahrussiam et al. [8], Nouveau et al. [12], Kong et al. [14], and Chayeuski et al. [15]. Such coatings reduce the adhesion of planing products to the surface of industrial planer knives, reduce the friction of the tool with the workpiece, and as limit the penetration of heat into the tools, radically extending its effective working life. These issues were described in detail in the works of Sheikh-Ahmad and Morita [16], Ratajski et al. [17], and Busuladzic et al. [18]. Currently, many types of coatings with single and multilayer structures produced by various types of chemical compounds are known. Some typical ones are presented in Table 1.

This study examined the influence of the developed coating on the durability and intensity of wear of industrial planer knife blades operating in production conditions compared to unmodified knives. Detailed analyses were carried out on industrial planer knives before and after processing for both groups of tools (modified and unmodified). The rounding radius and profile along the knife (size of worn edge displacement, wear area of the cutting edge) were determined using a stylus profilometer, while values of selected texture parameters of the rake surface of planer knives were measured using an optical profilometer. Additionally, visual analyses of the cutting edge were carried out using a digital microscope.

## 2. Process of Modification of the Operational Properties of Industrial Planer Knives

CrN/CrCN coatings were applied to TINA 900M device using the cathodic arc evaporation method from a chromium cathode of 99.99% purity and 100 mm diameter. The pressure and flow of gases (argon, nitrogen, and acetylene) were controlled using a Baratron capacitance head and MKS flow meters. Coatings were applied on planer knives made of HS6-5-2 steel with dimensions of 160 × 30 × 3 mm. As a reference, substrates of the same steel were mechanically polished to a roughness of approximately *Ra* ≈ 0.02 μm. After chemical cleaning, including an alkaline ultrasonic bath, the substrates were placed on a rotating table inside the vacuum chamber. CrCN/CrN coatings consisted of six CrCN + CrN modules, each about 400 nm thick, with the ratio of layer thickness in the module being 1:1. The total thickness of the coating was approximately 2.4 µm. The technology of PVD coating application has been presented in detail in earlier works [21,22,23,24,25].

## 3. Methodology of Experiments

**Figure 1:**
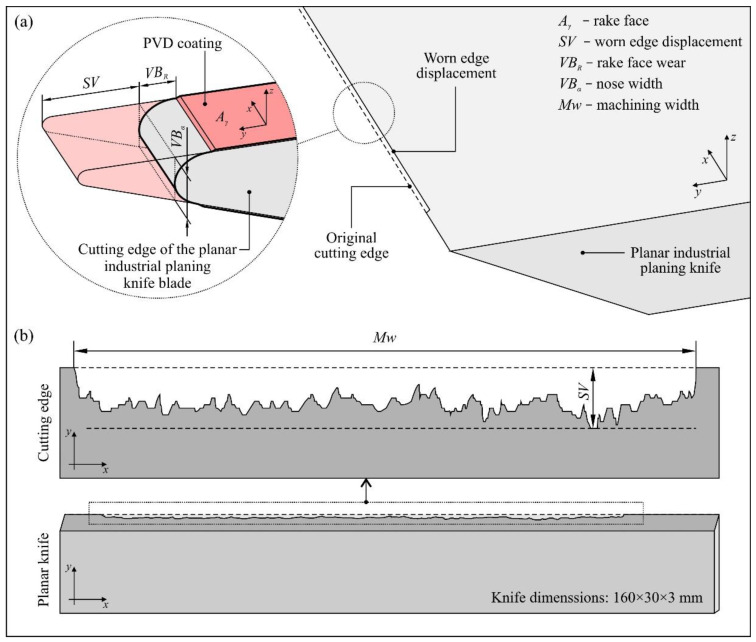
Graphical representation of (**a**) the cutting edge of the industrial planer knife blade with associated nomenclature; (**b**) basic elements of the machining width (*Mw*) and worn edge displacement (*SV*) of the cutting edge.

The tools with modified properties were mounted on cutter heads and tested under industrial conditions to determine their durability in relation to unmodified knives. Figure 1 presents graphical representation of the cutting edge of the industrial planer knife blade with PVD coating as well as basic nomenclature connected with the described issue.

### 3.1. Operational Tests under Production Conditions

For the experiments, a set of 18 industrial planer knives (dimensions: 160 × 30 × 3 mm, wedge angle: 40°, material: high-speed steel (HSS)) produced by Leitz GmbH & Co. KG (Oberkochen, Germany) was prepared. Six of them were coated with CrCN/CrN PVD coating. Table 2 presents the characteristics of planer knives used in the experiments and indicates their position in individual cutter heads.

**Figure 2:**
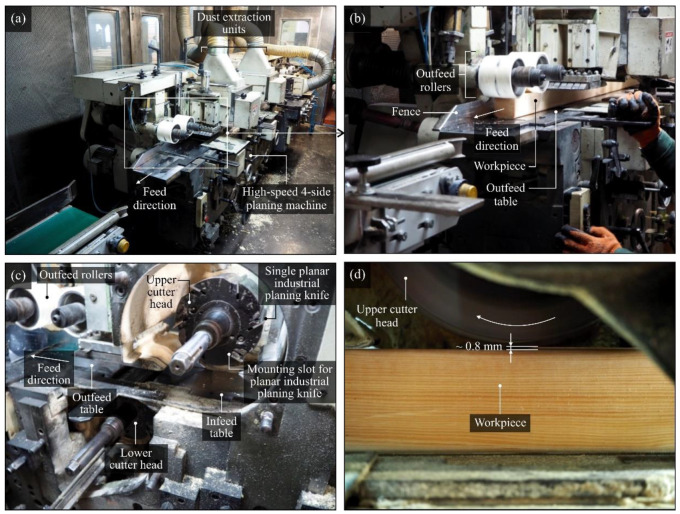
General view of (**a**) operational testing stand equipped with the high-speed four-sided planing machine Hydromat 22B, produced by Michael Weinig AG, during processing of the pine wood (Scots pine (*Pinus sylvestris* L.)); (**b**) calibration process on the first few elements of the production batch; (**c**) two cutter heads during assembly; (**d**) machining zone of the upper cutter head (U1) with visible machining allowance (in this case, ~0.8 mm), which was removed by the last set of industrial planer knives mounted on this head.

The planing process was carried out on a piece of wet pine (Scots pine (*Pinus sylvestris* L.) with 35–55% humidity) at feed speed of 57 m/min at a spindle speed of 6000 rpm. The total machining allowance was 5 mm, and it was removed for the most part by the first set of cutter heads in the roughing range. The tested knives were mounted on the second (last) set of heads, and the allowance for this operation was approximately 0.8 mm. In Figure 2, the operational testing stand equipped with the high-speed four-sided planing machine Hydromat 22B, produced by Michael Weinig AG (Tauberbischofsheim, Germany), is presented.

During the experiments, the modified industrial planer knives (mounted in L1) worked significantly longer than the unmodified industrial planer knives cooperating with them (mounted in U1). When a decision was made that the effective working life of the modified industrial planer knives had ended due to unsatisfactory quality of the lower surface of the workpiece, the upper surface planed by the second set of unmodified industrial planer knives still met the quality requirements. It should be noted that the process of preparing industrial planer knives for the planing process (filling, sharpening) as well as the operating conditions were identical for all tools used in the experiments.

### 3.2. Measurements of Rounding Radius, Worn Edge Displacement, and Wear Area of the Cutting Edge of Industrial Planer Knive Blades

**Figure 3:**
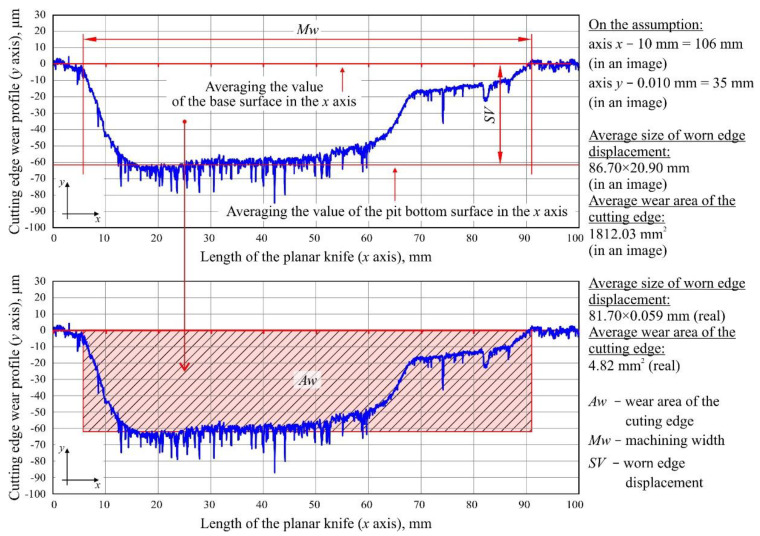
Method for determining the area of worn edge displacement of industrial planer knives using the example of a fragment of the surface profile (100 × 0.130 mm) of knife No. 9 (Set 1, Head U1).

**Figure 4:**
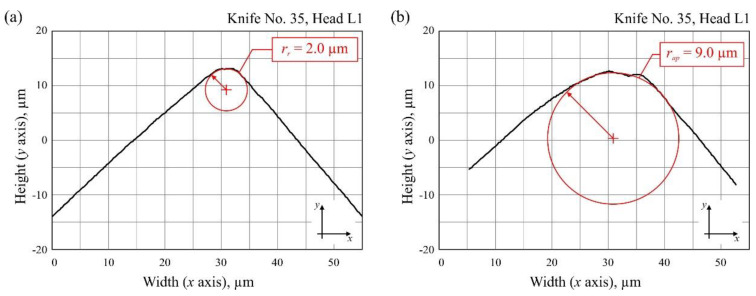
Method for determining the radius of the cutting edge of the industrial planer knives: (**a**) No. 35 (reference) (Set 1, Head L1); (**b**) No. 35 (after processing) (Set 1, Head L1).

The measurements were carried out using of a stylus profilometer (Hommel Tester T8000; Hommelwerke GmbH, Villingen-Schwenningen, Germany), which was more widely presented in the works of Kałonek et al. [29,30]. The instrument was equipped with a TKU-100 pick-up with a diamond stylus tip (opening angle: 90°, tip radius: 1.5 μm). The pick-up was part of a traverse unit (Waveline™ 60 Basic; tracing length: 60 mm, resolution: 0.1 μm, tracing speed: 0.1–3 mm/s) mounted on a measuring column (Wavelift™ 400M; maximum traverse: 400 mm). The column was, in turn, mounted on a granite plate (Wavesystem™ 780; dimensions: 780 × 500 × 100 mm). On the plate, a small angle vise was placed, and the industrial planer knife prepared for measurements was mounted on the clamps. To set the conditions and carry out measurements with the stylus profilometer, a Turbo Roughness 3.1 software (Hommelwerke GmbH, Villingen-Schwenningen, Germany) provided by the manufacturer of the instrument was used. The method for determining the area of worn edge displacement and the radius of the cutting edge of the planar industrial planer knives is presented in Figure 3 and Figure 4, respectively.

### 3.3. Measurement of the Texture of the Rake Face of the Industrial Planer Knives 

The measurements were carried out using an optical profilometer (Talysurf CLI 2000; Taylor-Hobson, Leicester, Great Britain) equipped with laser triangulation sensor LK-031 (Keyence Corp., Osaka, Japan), which allowed measurements in the range of 10 mm at resolutions of 1 μm (vertical) and 30 μm (lateral). The description of the general view of the instrument is given in detail in the works of Guo et al. [31], Elmas et al. [32], and Kapłonek et al. [33].

The measurement process conducted using Talyscan CLI 2000 2.6.1 software (Taylor-Hobson, Leicester, Great Britain) was carried out for the following parameters: area of a single topography (axes x, y, z): 4.80 × 4.80 × x.xx mm, number of profile points (axis x): 2401, distance between profile points (axis x): 2 μm, number of profiles (axis y): 321, and distance between profile points (axis y): 15 μm. 

All of the measurements were carried out in a nonworking zone. In the zone where the planing process took place, the wear effects of the cutting edge were visible. The surface characteristics of the industrial planer knives were analyzed using TalyMap Silver 4.1.2 (Digital Surf, Besançon, France). This dedicated surface analysis software allowed the calculation of selected surface texture parameters. The characteristics of the parameters used in this application are given in Table 3.

### 3.4. Microscopic Observation and Visual Analysis of the Cutting Edge Condition

**Figure 5:**
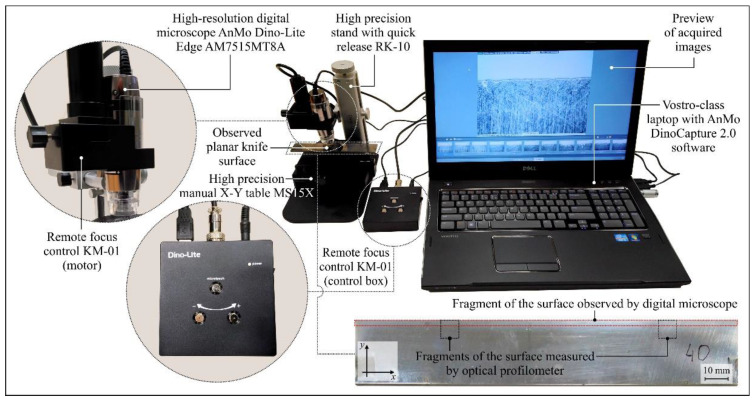
General view of the setup used for acquisition of digital images of selected fragments of the rake face of industrial planer knives based on the high-resolution digital microscope Dino-Lite Edge AM7515MT8A, produced by AnMo Electronics Corp.

The microscopic observations were carried out on a specially prepared setup, as shown in Figure 5. The main element of the setup was the high-resolution digital microscope AnMo Dino-Lite Edge AM7515MT8A, produced by AnMo Electronics Corp. (New Taipei City, Taiwan), equipped with a matrix complementary metal-oxide semiconductor (CMOS) detector providing 5 megapixel (2592 × 1944 pixel) resolution, which allowed observation of the surface of the industrial planer knives at magnification in the range of 700–900× as well as recording of a video sequence (30 fps). The microscope was powered via a signal cable connected to the USB port of a Vostro laptop produced by Dell Inc. (Round Rock, TX, USA). As a light source, a set of eight integrated light-emitting diodes (LEDs) characterized by the function of flexible control of illumination intensity (flexible LED control, FLC) was used. It allowed switching between brightfield and coaxial illuminations or combining them. Coaxial illumination technology is particularly beneficial because it reveals details that are difficult to observe under typical conditions. The instrument had an automatic magnification reading (AMR) function, which automatically detected the magnification and displayed it in the DinoCapture 2.0 software.

The image acquisition of the industrial planer knives was carried out for a selected fragment of the rake face before the machining process (as a reference) and a fragment of the rake face after the machining process with visible wear. The range of magnification was 690× to 700× (FOV 0.533 × 0.408 mm). All images were acquired in high resolution (2592 × 1944 pixels) and saved as *.TIF files.

## 4. Results and Discussion

This section presents the results of the experimental studies relating to the analysis of the following features:

- life of the knives (machining efficiency),
- radius of the cutting edge in and out of the working area,
- area of worn edge displacement,
- texture of the rake face of the industrial planer knives in and out of the working area, and
- condition of the cutting edge in both zones based on microscopic observations.

All analyses were carried out in relation to CrCN/CrN-coated knives (No. 35–40) and the first set of unmodified knives (No. 7–12). These sets of tools (cutter head U1 and L1) worked until the end of their shelf life, although their working time differed, thus providing a reliable basis for comparative analyses of the impact of the modifications on tool durability.

### 4.1. Life of the Knives

**Figure 6:**
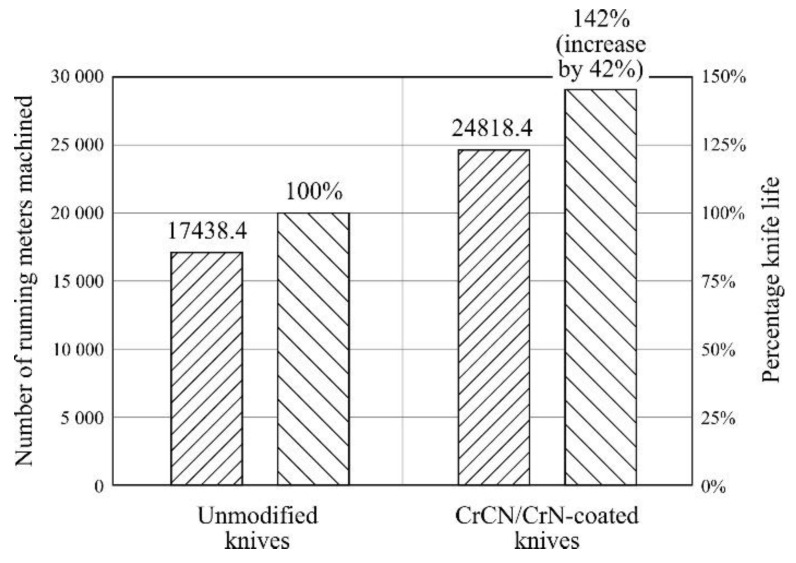
Comparison of the number of running meters of material machined using CrCN/CrN-coated and reference knives (unmodified) after operational tests carried out under industrial conditions.

Figure 6 shows a comparison of the number of running meters of material machined using CrCN/CrN-coated and reference knives (unmodified) after operational tests carried out under industrial conditions.

For the head with CrCN/CrN-coated knives, stable work and the desired surface quality of the machined surface was recorded for 24,818.4 running meters of solid pine wood beams (wet) with a cross section of 90 × 90 mm and a length of 2200 mm. However, the cutting head using unmodified knives worked effectively only up to the value of 17,438.4 running meters. This means that the modification of the cutting edge extended the shelf life of the knives by up to 142% compared to the quantity of machined wood (working time) done by the head equipped with unmodified knives. It should be emphasized that the end of the shelf life was decided arbitrarily by the planer operator on the basis of a sensory evaluation of the quality of the machined surface. Such an assessment, despite its subjectivity, is widely used in industrial conditions and depends to a large extent on the employee’s experience. The positive increase in the shelf life of the modified knives can be explained by the antiwear properties of the PVD coating.

### 4.2. Radius of Cutting Edge

**Figure 7:**
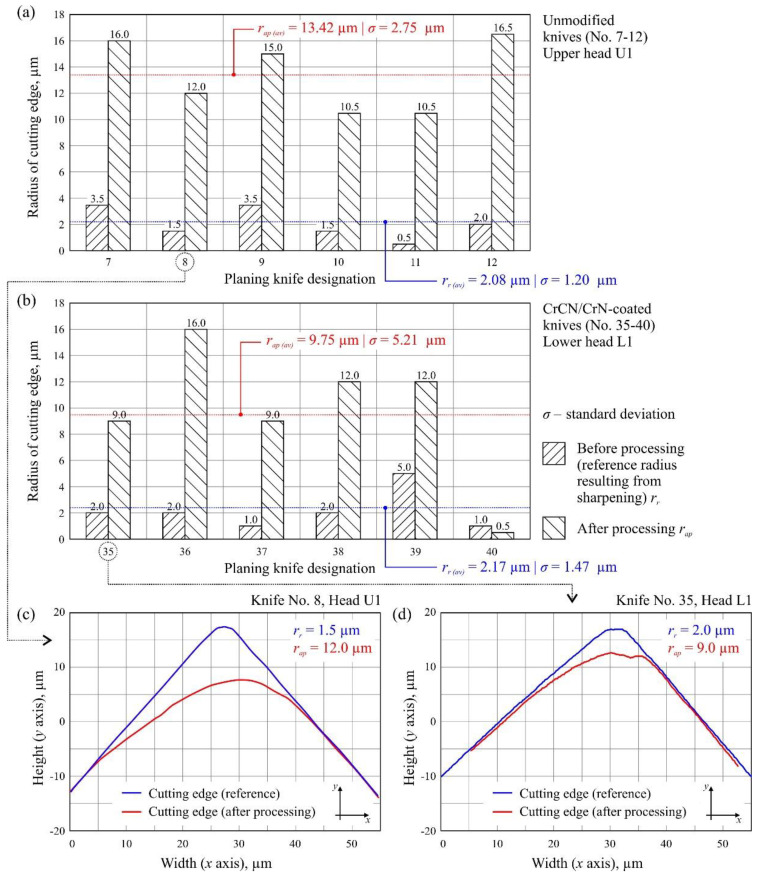
Measurement of the radius of the cutting edge of planer knives for (**a**) unmodified knives No. 7–12 and (**b**) CrCN/CrN-coated knives No. 35–40. Examples of specific values of the radius for (**c**) knife No. 8 (Set 1, Head U1) and (**d**) knife No. 35 (Set 1, Head L1).

Figure 7a,b presents a summary of the radius of the cutting edge in and out of the working area for all 12 analyzed knives as well as their mean values. Additionally, the wear of selected blade radii is presented in Figure 7c,d.

Analysis of the measured radii of planer knife blades indicated their significant differences, evaluated in relation to the reference radius (after sharpening) and the radius measured after the planing process. These differences ranged from 7× to 21× for unmodified knives and from 2× to 9× for CrCN/CrN-coated knives, with the radius increasing as a result of intensified blade wear. The average value of the radius of the cutting edge after sharpening was similar for both groups of tools under consideration, i.e., *r_r(av)_* = 2.08 µm for unmodified knives and *r_r(av)_* = 2.17 µm for CrCN/CrN-coated knives. Comparison of the average value of the radius of the cutting edge after processing for modified (*r_ap(av)_* = 9.75 µm) and unmodified knives (*r_ap(av)_* = 13.42 µm) at the end of their lives indicated a beneficial effect of CrCN/CrN coating.

### 4.3. Area of Worn Edge Displacement

**Figure 8:**
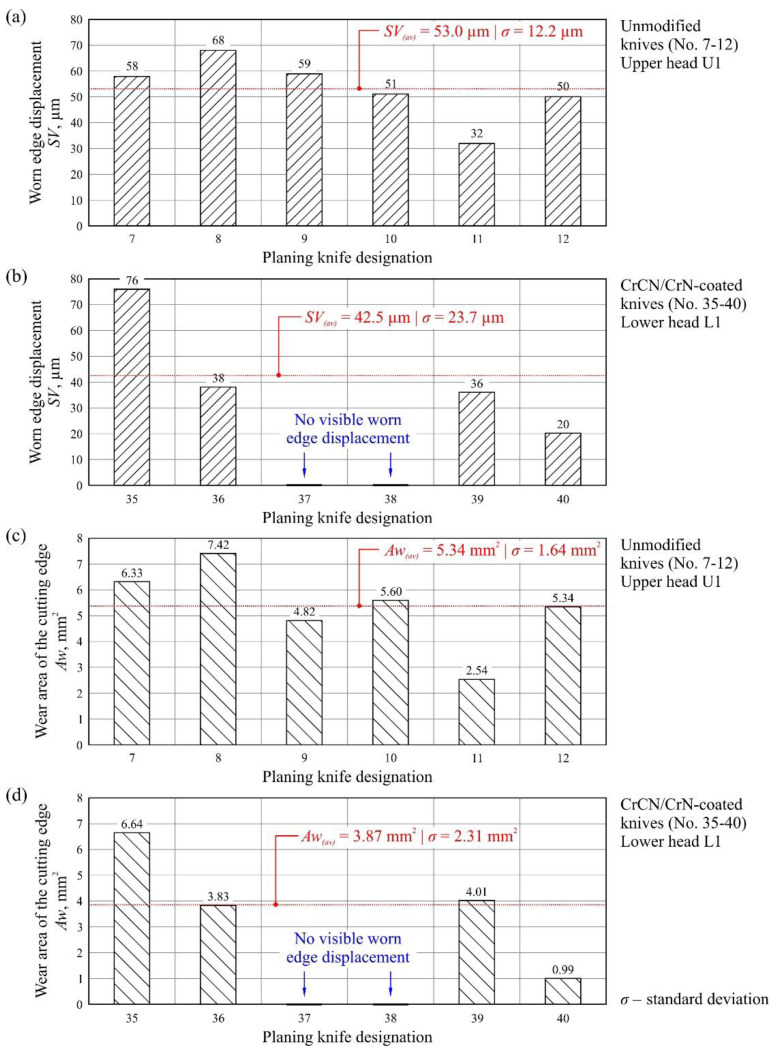
Measurement of the worn edge displacement (*SV*) of planer knives for (**a**) unmodified knives No. 7–12 and (**b**) CrCN/CrN-coated knives No. 35–40. Examples of specific values of wear area of the cutting edge for (**c**) unmodified knives No. 7–12 and (**d**) CrCN/CrN-coated knives No. 35–40.

In the next stage, the worn edge displacement (*SV*) as well as wear area of the cutting edge (*Aw*) were designated for unmodified and CrCN/CrN-coated knives. These measurements are shown in Figure 8, together with the average values of the parameters (*SV_(av)_* and *Aw_(av)_*) specified for each set of knives.

The measured values of worn edge displacement had comparable values for both modified (*SV* = 20–76 µm) and unmodified (*SV* = 32–68 µm) knives (Figure 8a,b). The values that were determined differed in relation to individual knives on a given cutter head by up to four times, e.g., *SV* = 20 µm for knife No. 40 and *SV* = 76 µm for knife No. 35 (Head L1, Figure 8b). Additionally, no clear worn edge displacement was identified for knives No. 37–38. For the wear area of the cutting edge directly from the measurement of *SV* and for the determined values (Figure 8c,d), similar dispersion was observed as that for the worn edge displacement.

The observed dispersion of the values of worn edge displacement and wear area between individual knives on a given cutter head was largely the result of differences in positioning of individual blades on the head as well as the varying degree of their deformation after removal, such as that caused by relaxation of stresses introduced into the structure of the tool due to the effect of heat in the planing process. The obtained results also indicated smaller values of both the parameters (*SV* and *Aw*) on the cutter head with mounted CrCN/CrN-coated knives. This could be clearly seen by comparing the average values of the analyzed parameters. They showed that the *SV_(av)_* was about 21% smaller and the *Aw_(av)_* was about 28% smaller for knives that had retained their cutting ability for a longer time thanks to the CrCN/CrN coating applied by PVD.

### 4.4. Texture of the Rake Face of Knives 

**Figure 9:**
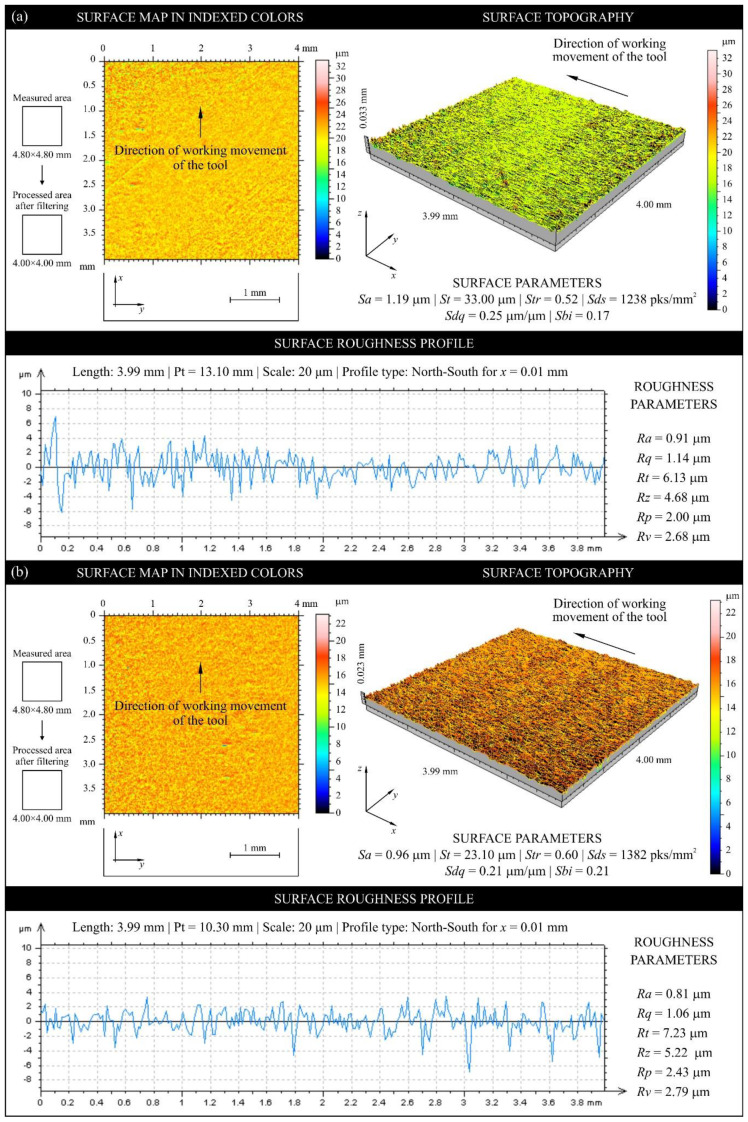
Collection of measurements obtained using an optical profilometer (Talysurf CLI 2000) for the rake face of unmodified industrial planer knife No. 9 (Set 1, Head U1) (**a**) before processing (reference) and (**b**) after processing.

**Figure 10:**
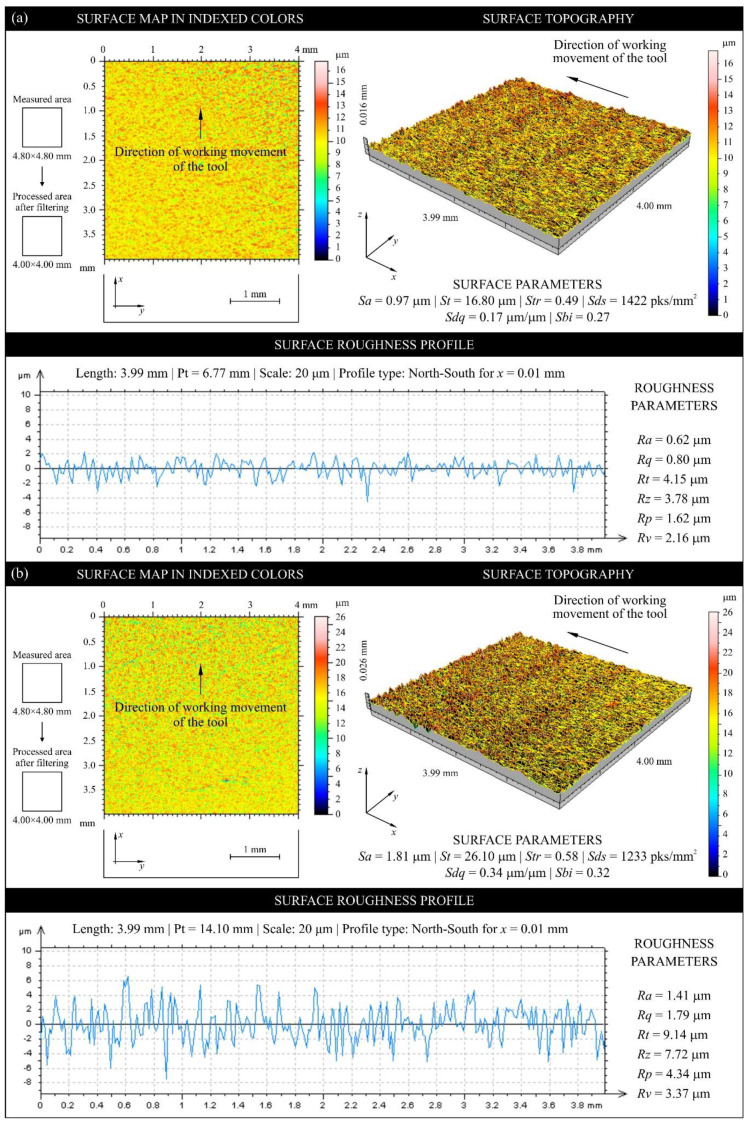
Collection of measurements obtained using an optical profilometer (Talysurf CLI 2000) for the rake face of CrCN/CrN-coated industrial planer knife No. 35 (Set 1, Head L1) (**a**) before processing (reference) and (**b**) after processing.

**Figure 11:**
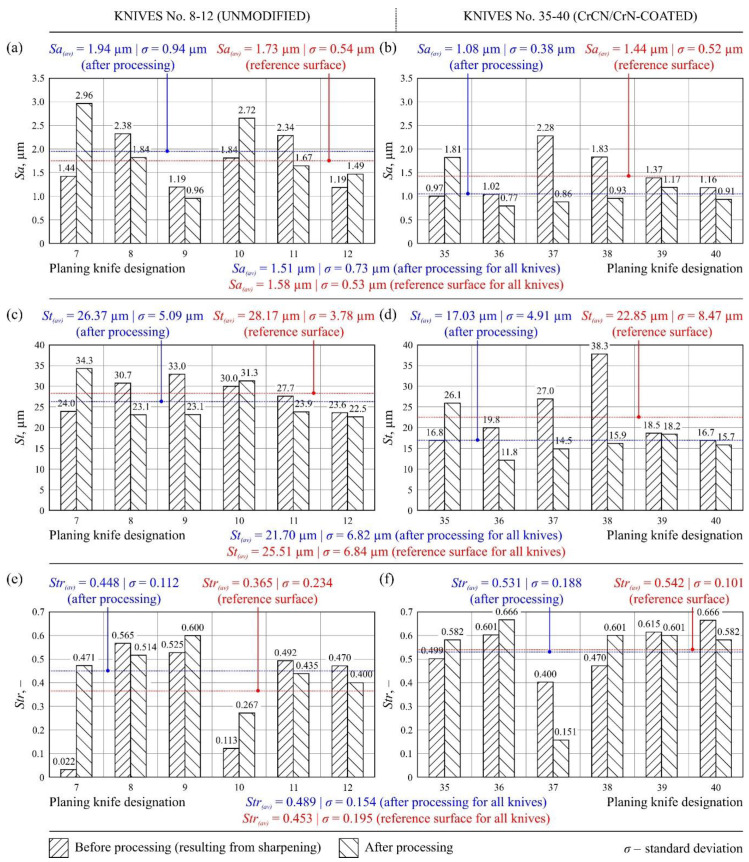
Values of texture parameters of the rake faces of (**a**,**c**,**e**) unmodified knives No. 7–12 and (**b**,**d**,**f**) CrCN/CrN-coated knives No. 35–40: (**a**,**b**) arithmetic mean deviation of the surface (*Sa*); (**c**,**d**) total height of the surface (*St*); (**e**,**f**) texture aspect ratio of the surface (*Str*).

**Figure 12:**
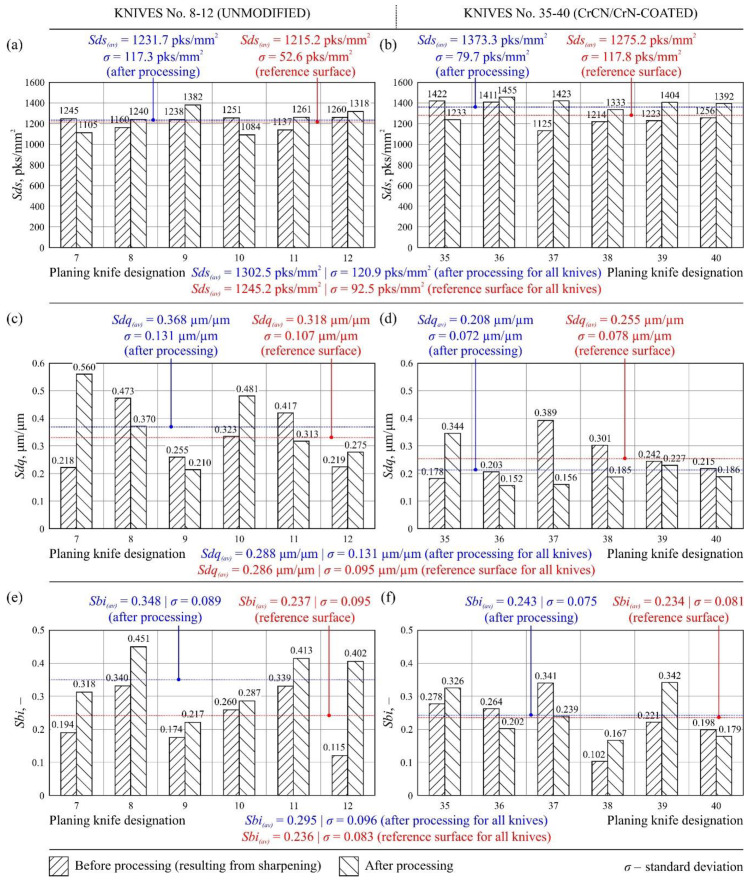
Values of texture parameters of the rake faces of (**a**,**c**,**e**) unmodified knives No. 7–12 and (**b**,**d**,**f**) CrCN/CrN-coated knives No. 35–40: (**a**,**b**) density of summits of the surface (*Sds*); (**c**,**d**) root-mean-square slope of the surface (*Sdq*); (**e**,**f**) surface bearing index (*Sbi*).

Figure 9 shows sample texture measurements of the rake face of the unmodified industrial planer knife (knife No. 9, Head U1) in unused and worn zone, while Figure 10 shows the same compression results for the tool modified with PVD coating (knife No. 35, Head L1). Figure 11 and Figure 12 show diagrams comparing the values of all analyzed texture parameters of the surface in the worn zone and, as a reference, the values determined in the part of the edge that did not take part in the planing process for all knives from U1 (No. 7–12) and L1 (No. 35–40) heads.

The analysis of the six parameters of surface texture, representing groups of amplitude, spatial, hybrid, and functional parameters, made it possible to properly characterize the state of the cutting edge surface of the planer knives that were worn out as a result of machining and those that did not take part in planing. A comparison of the mean values of parameters *Sa_(av)_*, *St_(av)_*, *Str_(av)_*, *Sds_(av)_*, *Sdq_(av)_,* and *Sbi_(av)_*, presented in Figure 11 and Figure 12, made it possible to state that the parametric evaluation of the rake face texture did not differ significantly for unmodified and CrCN/CrN-coated knives.

The *Sa* parameter, defined as the arithmetic mean deviation of the surface, is the basic amplitude parameter used in the spatial evaluation of machined surface texture. The average value of this parameter for all analyzed knives in the reference zone was *Sa_(av)_* = 1.58 μm, while it was *Sa_(av)_* = 1.51 μm in the zone after processing (Figure 11a,b). For most of the analyzed knives, a decrease in this parameter was recorded on the surfaces after work. The lower value of *Sa* in the zone after work was largely determined by abrasive wear. It caused smoothing of the vertices of the rake face, which had formed an uneven shape during the knife manufacturing process. The measurements also showed that the average values of *Sa* were significantly lower in the case of CrCN/CrN-coated knives, showing the influence of PVD coating on the measured surface roughness of the knives.

Analogous conclusions can be drawn from the analysis of the total height of the surface (*St*). This is another amplitude parameter, whose average values for both the considered zones of all analyzed tools were *St_(av)_* = 21.70 μm (zone after processing) and *St_(av)_* = 25.51 μm (reference zone), as shown in Figure 11c,d. However, the measurements of both the amplitude parameters (*Sa* and *St*) were characterized by significant dispersion, often exceeding 200% in a set of tools working together on one head (Figure 11a–d). This might have been due to the heterogeneity of the evaluated surfaces, which were subject to different wear and degradation of the rake face during long-term use.

The texture aspect ratio of the surface (*Str*) can be used to determine the machining traces in any direction of the analyzed surface. The average values of this basic spatial parameter for both zones under consideration of all the analyzed tools were *Str_(av)_* = 0.489 μm (zone after processing) and *Str_(av)_* = 0.453 μm (reference zone), as shown in Figure 11e,f. In addition, significant variability was clearly visible in the values of this parameter for individual knives. On the basis of the *Str* values obtained, it can be concluded that there was no change in the direction of the machining marks on the surface of the analyzed knives as a result of machining.

The second additional spatial parameter applied was *Sds*, describing the density of the surface summits (number of summits per unit area, given in mm^2^ in this case). Its average values for both zones under consideration of all the analyzed tools were *Sds_(av)_* = 1302.5 pks/mm^2^ (zone after processing) and *Sds_(av)_* = 1245.2 pks/mm^2^ (reference zone), as shown in Figure 12a,b. The values showed no significant differences in density of surface summits, both between modified and unmodified knives as well as changes resulting from knife wear.

The additional hybrid parameter, *Sdq*, defined as the root-mean-square slope of the surface, was used to measure the slopes that constitute the surface and could be useful in identifying surfaces with similar average unevenness. With few exceptions, the values of *Sdq* decreased as a result of knife operation (Figure 12c,d). However, the mean values of the slope of the surface for both zones of all the analyzed tools were very similar, i.e., *Sdq_(av)_* = 0.288 μm/μm (zone after processing) and *Sdq_(av)_* = 0.286 μm/μm (reference zone), as shown in Figure 12c,d. Comparison of mean values for the group of unmodified and CrCN/CrN-coated knives showed about 30% reduction of *Sdq* for modified knives, which might have been a result of PVD coating.

The functional *Sbi* (surface bearing index) parameter of auxiliary nature contained the following average values for all analyzed tools: *Sbi_(av)_* = 0.295 (zone after processing) and *Sbi_(av)_* = 0.236 (reference zone), as shown in Figure 12e,f. The parameter *Sbi* refers to the surface load capacity, and it can be concluded that the application of CrCN/CrN antiwear coating did not significantly affect this aspect of surface texture evaluation for the analyzed rake faces of planer knives. However, an unambiguous evaluation of *Sbi* was difficult due to significant dispersion of the determined surface bearing index values (Figure 12e,f).

### 4.5. Microscopic Observation and Visual Analysis of the Cutting Edge Condition

**Figure 13:**
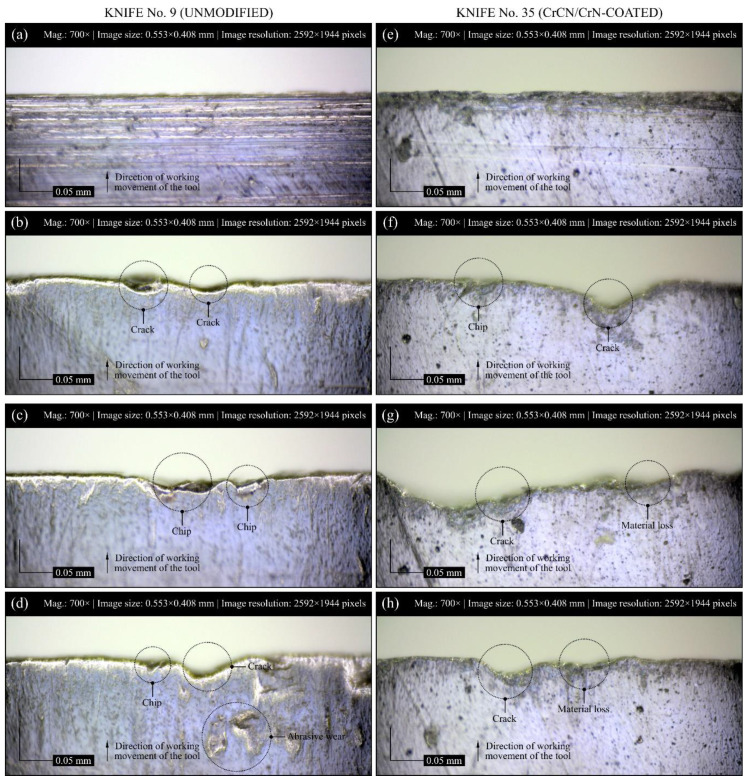
Selected results of digital images acquired of the rake face of industrial planer knives: (**a**) surface of unmodified knife No. 9 (Set 1, Head U1) (reference), (**b**–**d**) with various visible forms of surface wear; (**e**) modified knife No. 35 (Set 1, Head L1) (reference), (**f**–**h**) with visible wear of the surface.

In addition to measurements with the stylus profilometer (Hommel-Teser T8000 from Hommelwerke) and optical multiprophilometer (Taylor-Hobson CLI 2000) of cutting edge radius in and out of the working area, microscopic observations and acquisition of digital images of the cutting edge in the pre- and postmachining conditions were also carried out to analyze the worn edge displacement of industrial planer knives and their geometric texture. Selected results of the acquisition process for modified knife No. 35 (Set 1, Head L1) and unmodified knife No. 9 (Set 1, Head U1) are presented in Figure 13a–d and Figure 13e–h, respectively. Additionally, the form of wear and the level of wear intensity occurring on the rake face of all the analyzed industrial planer knives are presented in Table 4.

Observations of the tool surfaces, regardless of whether or not they concerned modified or unmodified knives, revealed numerous differences, resulting mainly from various forms of wear and their different intensities. Microscopic observations provided the basis for qualitative evaluation of the cutting edge after work, and they showed a limited number of wear forms and lower intensity of wear on CrCN/CrN-coated knives compared to unmodified knives (Table 4). The rake face of the unmodified knives was highly dominated by abrasive wear, cracks, and chips, with a slight share of the initial lapping, while chips were the main form of wear observed on the rake face of CrCN/CrN-coated knives. On these knives, abrasive wear and cracks were occurring at a slightly lesser extent, with a total lack of wear arising from the initial lapping. A small number of wear forms and low intensity of wear correlated with the relatively lower values of the surface texture parameters measured using the optical profilometer for CrCN/CrN-coated knives (Figure 11 and Figure 12). This confirmed the beneficial effect of the implemented tool modification. The PVD coating made it possible to significantly reduce the intensity of the wear process. When evaluating results of the observation, it should be noted that the modified knives worked much longer (142% increase in durability) than the unmodified ones.

### 4.6. Summary of Test Results

Table 5 shows a synthetic summary of the most important results of analyses conducted on the planer knives after pine wood planing process was carried out under industrial conditions. The measurements and analyses unequivocally indicate the possibility of significant reduction in the intensity of wear by applying a CrCN/CrN coating to the planer knives using the PVD method. An extremely important aspect of this research is the utilitarian potential. The tests were conducted in industrial conditions taking into account the technological regime of a medium-sized wood processing plant. This means that all disturbances resulting from industrial practice took place, which is significantly different from laboratory conditions. The effect of interference is particularly evident when comparing the values and standard deviation *σ* of the individual averages (Table 5). In some cases, the standard deviation, which is a measure of the scattering of quantities around the expected value, was even about 50% of the average value. In conclusion, it can be stated that the favorable research results and the adopted research methodology brings the developed solution significantly closer to industrial implementation.

## 5. Conclusions

In this study, a wide set of analyses allowed a comprehensive and complementary assessment of cutting edge wear during planing of wet pine wood. The wear phenomena and their intensity differed depending on the tested group of tools (modified and unmodified), leading to significant differences in the life of both these groups. Below are the most important conclusions resulting from the research.

1. Comparison of the volume of material removed by a set of heads with modified industrial planer knives and heads equipped only with unmodified knives indicated an increase in durability of up to 142% when using CrCN/CrN-coated tools.
2. Analysis of the results obtained by measuring the radius of the cutting edge in and out of the work area showed significant variation, evaluated in relation to the reference radius and the radius measured after the operation of the blade in the planing process. The difference in the radii was much higher for unmodified industrial planer knives (ranging from 7× to 21×) than the CrCN/CrN-coated knives (ranging from 2× to 9×).
3. Comparison of the average value of the cutting edge radius after processing for modified (*r_ap (av)_* = 9.75 µm) and unmodified industrial planer knives (*r_ap (av)_* = 13.42 µm) indicated beneficial effect of CrCN/CrN coating.
4. Analysis of the results obtained during measurements of the area of worn edge indicated significant dispersion of the depth of edge displacement (*SV*) as well as the wear area (*Aw*). These might have resulted from issues such as inaccuracies in blade positioning on the planing head as well as varying degrees of deformation after removal caused by relaxation of stresses introduced into the tool structure as a result of the heat generated during the planing process.
5. Smaller values of edge displacement (*SV*) as well as wear area (*Aw*) were observed on the cutter head mounted with CrCN/CrN-coated knives (*SV_(av)_* was about 21% smaller and *Aw_(av)_* was about 28% smaller) than in the case of unmodified knives.
6. Analysis of the results obtained during the parametric evaluation of the texture of the rake face of industrial planer knives allowed us to conclude that this assessment did not significantly differ for unmodified and CrCN/CrN-coated blades.
7. For all analyzed surfaces of the rake faces of industrial planer knives, it was concluded that the basic parameters of their surface texture, especially *Sa* and *St*, were clearly decreasing in the zone after work. This was a result of abrasive wear as it smoothed the vertices of the rake face, which had formed an uneven shape during the knife manufacturing process.
8. Measurements of the surface texture also allowed us to conclude that the process of PVD coating (in the case of modified knife blades) did not significantly affect the roughness of the rake face.
9. Microscopic observations of industrial planer knife blades (unmodified and CrCN/CrN-coated) after work made it possible to determine the usual occurrence of several different forms of wear at the same time (abrasive wear, breakage, crushing), characterized by different sizes and distributions on the analyzed surface. For CrCN/CrN-coated industrial planer knife surfaces, a relatively less intensification of wear was observed, which correlated with lower values of optically measured surface texture parameters.

## Figures and Tables

**Table 1:** Selected typical coatings with single and multilayer structures.

Coating(s)	Layer		References
	Single	Multi	
CrN	+		Chekour et al. [19], Faga and Settineri [20], Gilewicz et al. [21], Kong et al. [14]
Cr_2_N/CrN		+	Warcholiński et al. [22]
CrCN/CrN		+	Kong et al. [14], Szymański et al. [23], Gilewicz et al. [24], Krela et al. [25], Warcholiński and Gilewicz [26]
CrCN/CrN + ta-C		+	Gilewicz et al. [24]
ZrN, Mo + Mo_2_N	+	+	Kuleshov et al. [27]
TiCN/TiCN + ZrN		+	Siow et al. [28]
TiAlN, TiAlN/TiN	+	+	Fahrussiam et al. [8], Warcholiński and Gilewicz [26]
TiAlN/TiSiN		+	Fahrussiam et al. [8]
TiAlN/TiBON		+	Fahrussiam et al. [8]

**Table 2:** Characteristic of industrial planer knives used in the experiments.

Set No.	Cutter Head	M ^1)^	UM ^2)^	Designation of Planer Knives
**1**	U1 and U2 (upper head)	–	Yes	Initially equipped with knives No. 7–12 (U1), and after they had worn out, replaced with another set of knives No. 13–18 (U2)
	L1 (lower head)	Yes	–	No. 35–40

^1)^ M, modified industrial planer knives; ^2)^ UM, unmodified industrial planer knives.

**Table 3:** Surface texture parameters used for characterization of the surface of industrial planer knives.

Group	Parameter	Unit	Description	Importance	IOP	CMS
Amplitude ^1^	*Sa*	μm	Arithmetic mean deviation of the surface	Essential	Yes	Yes
	*St*	μm	Total height of the surface	Essential	Yes	Yes
Spatial ^1^	*Str*	–	Texture aspect ratio of the surface	Essential	Yes	Yes
	*Sds*	pks/mm^2^	Density of summits of the surface	Additional	–	–
Hybrid ^1^	*Sdq*	μm/μm	Root-mean-square slope of the surface	Additional	–	No
Functional ^1^	*Sbi*	–	Surface bearing index	Additional	No	No

^1^ Parameters are included in the ISO 25178-2:2012 standard [34] and EUR 15178 EN report [35]; IOP, influence on the operational properties; CMS, control possibility in the manufacturing process.

**Table 4:** Forms of wear observed on the rake face of the analyzed industrial planer knives.

Set No.	Cutter Head	Planer Knife Designation	Form of the Wear			
			Initial Lapping	Abrasive Wear	Crack	Chip
**1**	**U1** **(Upper head)** **unmodified knives**	7		++	++	++
		8	+	++	++	++
		9		+++	++	++
		10		++	++	+
		11	++	+	+	
		12	++	+	+	+
	**L1** **(Lower head)** **CrCN/CrN-coated knives**	35			+	++
		36			+	++
		37			+	+++
		38				+
		39		++	++	++
		40		+	+	+

Surface wear intensity: +++ high, ++ moderately, + slight.

**Table 5:** Synthetic summary of the most important results of analyses conducted on the tested industrial planer knives.

Knife Type		Unmodified	CrCN/CrN-Coated
Number of running meters processed		17,438.4	24,818.4
Percentage increase in knife life		100%	142%
Average radius of cutting edge before processing *r_r (av)_*		2.08 µm *σ* = 1.20 µm	2.17 µm *σ* = 1.47 µm
Average radius of cutting edge after processing *r_ap (av)_*		13.42 µm *σ* = 2.75 µm	9.75 µm *σ* = 5.21 µm
Average worn edge displacement *SV_(av)_*		53.0 µm *σ* = 12.2 µm	42.5 µm *σ* = 23.7 µm
Average area of worn edge displacement *Aw_(av)_*		5.34 mm^2^ *σ* = 1.64 mm^2^	3.87 mm^2^ *σ* = 2.31 mm^2^
Texture parameters of the rake face of knives after processing	*Sa_(av)_*	1.94 µm *σ* = 0.76 µm	1.08 µm *σ* = 0.38 µm
	*St_(av)_*	26.37 µm *σ* = 5.09 µm	17.03 µm *σ* = 4.91 µm
	*Str_(av)_*	0.448 *σ* = 0.112	0.531 *σ* = 0.188
	*Sds_(av)_*	1231.7 pks/mm^2^ *σ* = 117.3 pks/mm^2^	1373.3 pks/mm^2^ *σ* = 79.7 pks/mm^2^
	*Sdq_(av)_*	0.368 μm/μm *σ* = 0.131 μm/μm	0.208 μm/μm *σ* = 0.72 μm/μm
	*Sbi_(av)_*	0.348 *σ* = 0.089	0.243 *σ* = 0.075
Forms of wear		Initial lapping, abrasive wear, crack, chip	Abrasive wear, crack, chip
Wear intensity		Moderate (++) and high (+++)	Slight (+) and moderate (++)

*σ*: standard deviation.

## Nomenclature

AMR	automatic magnification reading
CMOS	complementary metal-oxide semiconductor
CrCN	chrome carbon nitride
CrN	chromium nitride
CVD	chemical vapor deposition
FLC	flexible LED control
FOV	field of view (mm)
HSS	high-speed steel
LED	light-emitting diode
PVD	physical vapor deposition
M	modified industrial planer knives
ta-C	tetrahedral carbon
TiAlN	titanium aluminum nitride
TiBON	titanium boron oxide nitride
TiCN	titanium carbon nitride
TiN	titanium nitride
TiSiN	titanium silicon nitride
UM	unmodified industrial planer knives
ZrN	zirconium nitride
*r_ap_*	radius of the cutting edge (after processing) (μm)
*r_r_*	radius of the cutting edge (reference) (μm)
*Aγ*	rake face of the industrial planer knife
*Aw*	wear area of the cutting edge (mm^2^)
*Mw*	machining width (mm)
*Sa*	arithmetic mean deviation of the surface (μm)
*Sbi*	surface bearing index
*Sdq*	root-mean-square slope of the surface (μm·μm^−1^)
*Sds*	density of summits of the surface (pks/mm^2^)
*St*	total height of the surface (μm)
*Str*	texture aspect ratio of the surface
*SV*	worn edge displacement (μm)
*VB_α_*	nose width (μm)
*VB_R_*	rake face wear (μm)
*σ*	standard deviation
